# Dr. Allport Dead

**Published:** 1893-04

**Authors:** 


### DR. ALLPORT DEAD.

Dr. W. W. Allport died at his residence in Chicago, March 21, after a prolonged attack of erysipelas.

We have not the space to do more than notice this important and sad event, but will give a full account of his life-work in the May number.

